# Eco-friendly approaches to phytochemical production: elicitation and beyond

**DOI:** 10.1007/s13659-023-00419-7

**Published:** 2024-01-10

**Authors:** Kritika Jalota, Vikas Sharma, Chiti Agarwal, Suruchi Jindal

**Affiliations:** 1 Division of Molecular Biology and Genetic Engineering, School of Bioengineering and Biosciences, Lovely Professional University, Phagwara, 144411 India; 2 Washington State University, Pullman, USA

**Keywords:** In-vitro system, Abiotic/biotic elicitors, Plant secondary metabolites, Metabolic engineering, CRISPR-Cas

## Abstract

Highly ameliorated phytochemicals from plants are recognized to have numerous beneficial effects on human health. However, obtaining secondary metabolites directly from wild plants is posing a great threat to endangered plant species due to their over exploitation. Moreover, due to complicated structure and stereospecificity chemical synthesis of these compounds is a troublesome procedure. As a result, sustainable and ecofriendly in vitro strategy has been adopted for phytochemicals production. But, lack of fully differentiated cells lowers down cultured cells productivity. Consequently, for enhancing yield of metabolites produced by cultured plant cells a variety of methodologies has been followed one such approach includes elicitation of culture medium that provoke stress responses in plants enhancing synthesis and storage of bioactive compounds. Nevertheless, for conclusive breakthrough in synthesizing bioactive compounds at commercial level in-depth knowledge regarding metabolic responses to elicitation in plant cell cultures is needed. However, technological advancement has led to development of molecular based approaches like metabolic engineering and synthetic biology which can serve as promising path for phytochemicals synthesis. This review article deals with classification, stimulating effect of elicitors on cultured cells, parameters of elicitors and action mechanism in plants, modern approaches like metabolic engineering for future advances.

**Graphical Abstract:**

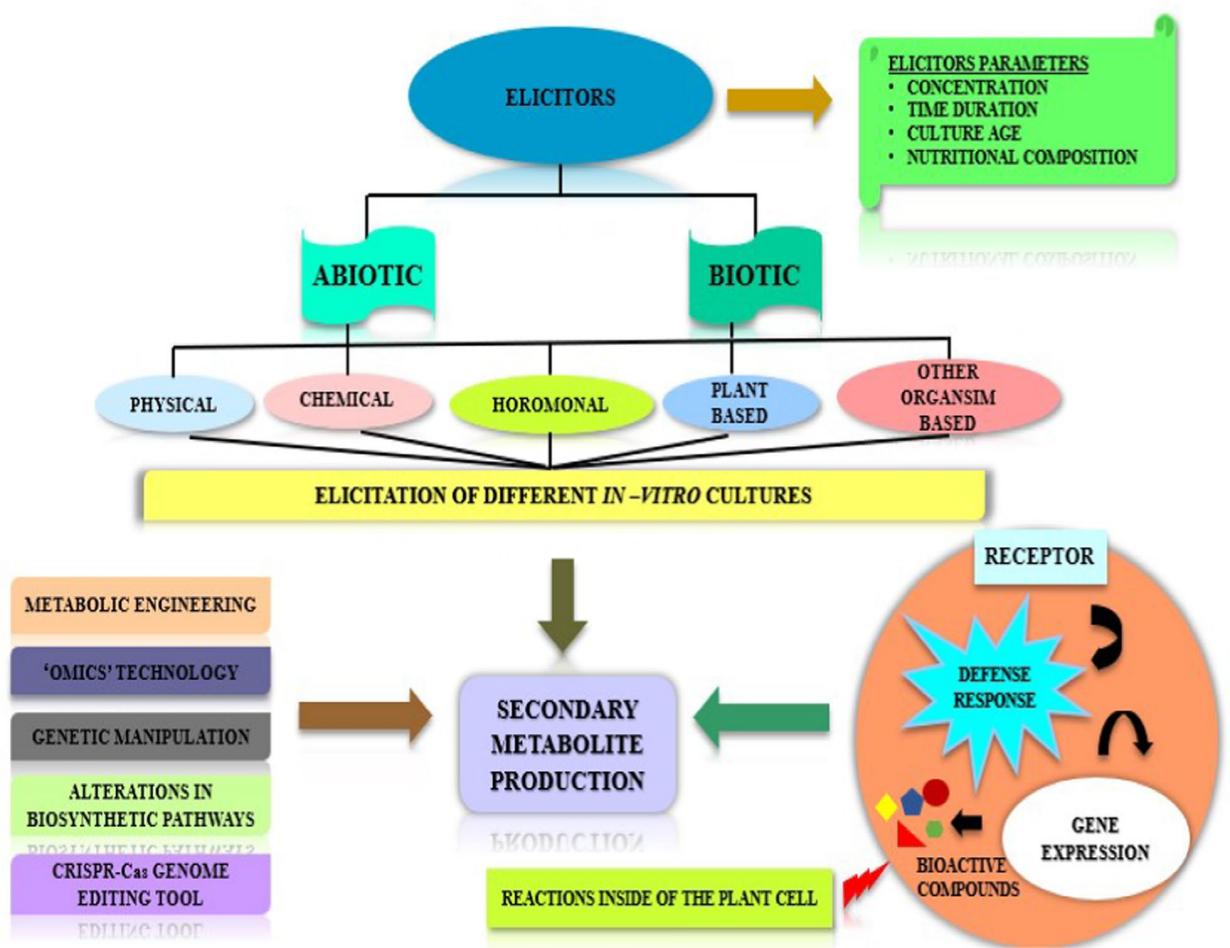

## Introduction

**Fig. 1:**
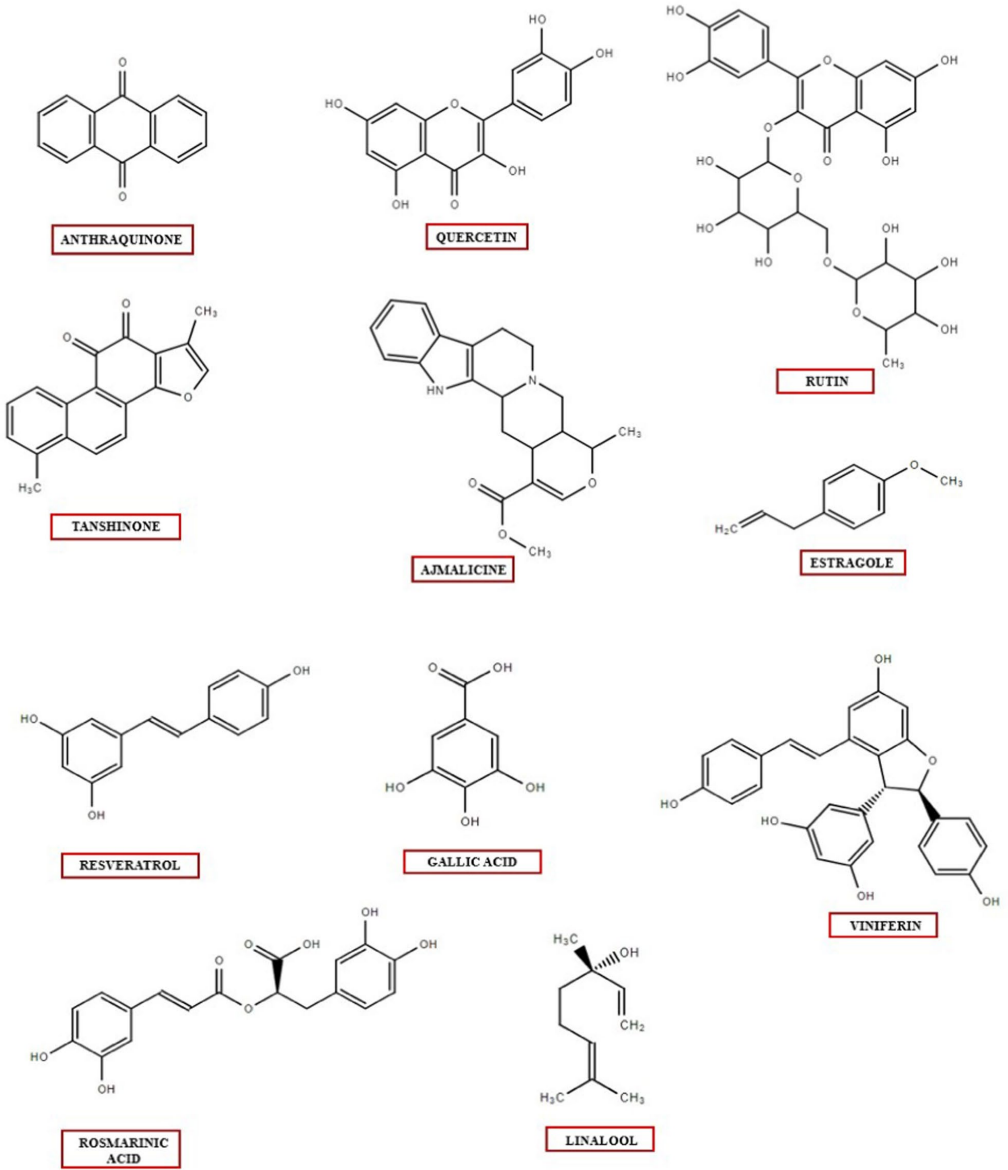
The chemical structures of plant secondary metabolites

Since a long time, plants are serving as an important source in meeting all the necessities of human beings such as fuel, shelter, food etc. because of the availability of numerous compounds in plants termed as plant bio actives. Compounds such as lipids, carbohydrates, amino acids are produced in large amounts and are responsible for the structure of plants hence termed as primary metabolites. Besides this a wide range of other compounds are synthesized in minute quantities in plants called secondary metabolites to carry out their physiological roles by enabling them to stand firm against unfavorable environmental conditions. Stress signals like deficiency of nutrients, attack by pathogenic microbes, environmental factors are responsible for the production of bio actives as a defense response by the plant system. Moreover, these compounds own biological activity averse to microbes, responsible for signaling and defense responses of the plants. Albrecht Kossel for the first time gave the concept of plant secondary metabolites [1]. Bio actives or phytopharmaceuticals include volatile oils, alkaloids, terpenoids, phenols, glycosides etc. Secondary metabolites are classified into four major categories that is terpenes, phenolics, and Nitrogen and Sulphur containing compounds [2, 3]. Figure 1 provides chemical structures of commonly produced plant secondary metabolites within the plant system. They are essential due to their utilization as precautionary or remedial medicines for certain diseases. These compounds are being used in aromatics, cosmetics industry, pharma industry, agrochemicals, food additives etc. [1]. The growing benefits of secondary metabolites from last a few years has inculcated interest in exploring more about secondary metabolism, especially in the feasibility of making alterations in phytochemical synthesis [4].

However, production level of plant bioactive compounds is considerably low (< 1%) and mainly determined by growth as well as physiological stages of the plant. Moreover, major complexity lies in the fact that plants, being a living entity, possess inherent biological variability. The chemical content of plant products shows variation even if plant material originates from the same plant species. Lack of repeatability is mainly because of two major reasons, variation in genetic makeup and different environmental conditions in which they grow [4]. Further, due to ecological, political or geographical factors, there is an increasing shortage in supply of the plant raw materials from which these bio actives are produced. Furthermore, the metabolite formation is many a times confined to a specific plant species or genus and are produced mainly at a particular growth and developmental stage, or under certain unfavorable environmental conditions or nutrient deficient situations. Due to troublesome cultivation, plants are being taken from field, which consequently threatens the survival of the plant species putting them on verge of getting extinct. Moreover, growth rate of certain plant species is very slow. As a result, there is a difficulty in fulfilling the market demands for required compounds even after proper planning. Further, it is challenging and economically not feasible to extract fine chemical compounds from plants and yield is also poor. Due to all these factors substantial attempts have been done in the field of biotechnology for synthesizing plant products using in-vitro culture techniques [5].

Culturing of plant tissue has become a favorable approach for the continual and commercial level synthesis of secondary metabolites. There are numerous benefits of using plant tissue culture such as it is independent of the environmental or geographic conditions, production and the quality can be regulated, life cycle of plant gets shortened in comparison to the field growing plant and escapes the use of land resources. In fact, from over a decade, some plant bio actives like shikonin, ginseng saponins and paclitaxel have been synthesized using this methodology [6]. Besides this, many studies have been targeted on strategies that enhances plant in-vitro cultures productiveness, which includes optimization of media, cell line selection, cell immobilization, precursor feeding and elicitation. Out of these manipulating approaches, using elicitor is considered to be a very appealing method to be used in increasing formation of plant natural product. Current studies have revealed that higher yield of pharmaceutically valuable components can be achieved by treating plant cultures with external stimuli or elicitors [7]. The use of living or non-living elicitors in stimulating the formation of highly-value added compounds has become an effective approach and is being used in decreasing the process time essential in accomplishing high product concentrations and enhanced volumetric productivity [8]. Besides this useful approach of elicitation, with technological advancement molecular based techniques such as metabolic engineering, genome editing tools etc. are gaining popularity in improving the formation along with accumulation of plant valuable compounds [9]. Therefore, present study is compilation of different types of stressors employed in culture system for improving culture growth and production of plant secondary products. Furthermore, we have tried revealing action mechanism along with the futuristic advancement and scope.

## Classification and impact

**Fig. 2:**
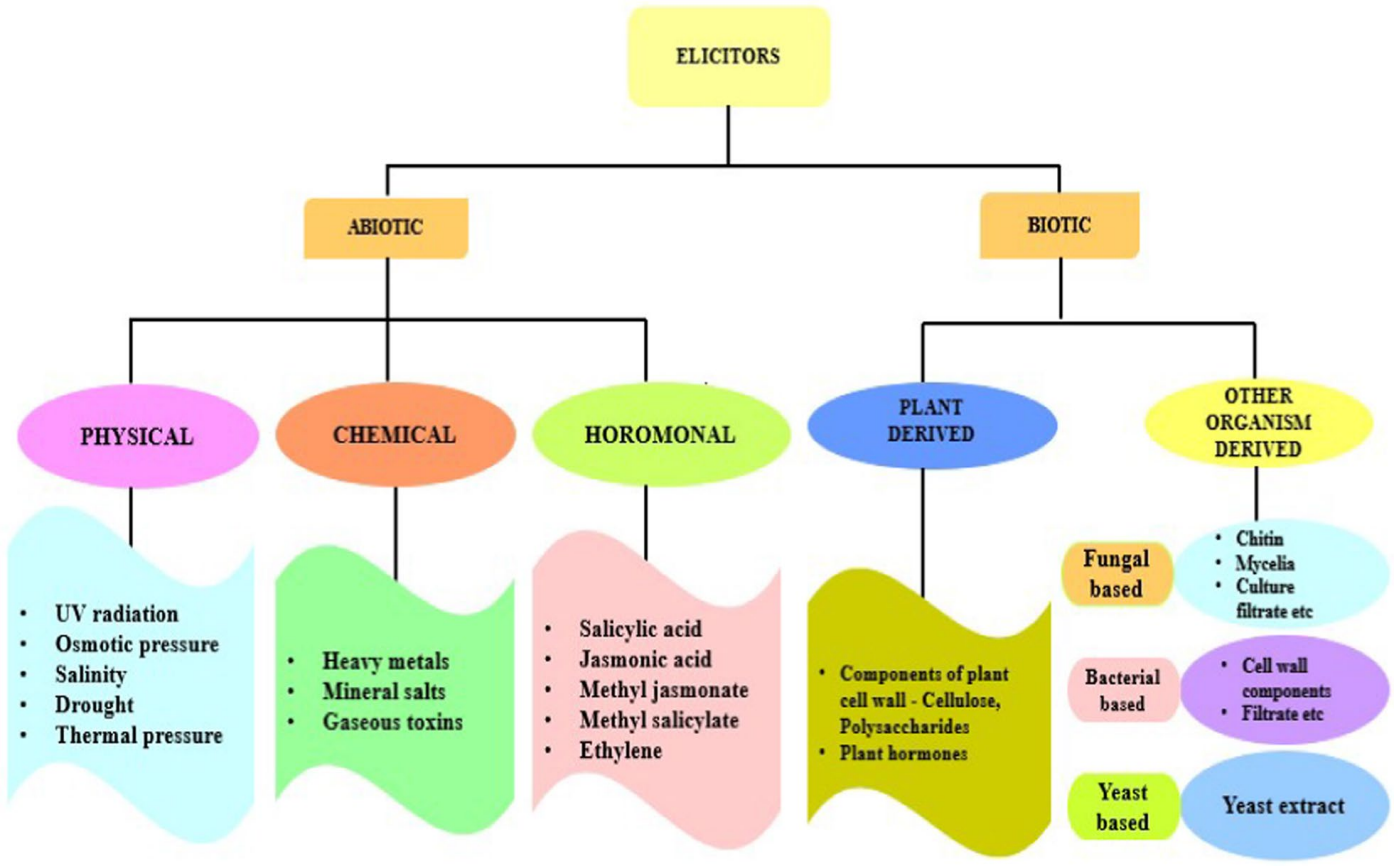
Illustrating flow chart of the classification of elicitors

Elicitors are those compounds that can stimulate any kind of physiological deformity of plant encouraging secondary metabolism for the safety of the whole plant [5, 10]. Elicitors can be categorized into two groups based upon their nature namely, Biological and non-biological [5]. The biotic stressors are the ones having biological origin, obtained either from the microbial sources such as fungi, bacteria, virus, from herbivore infection (exogenous elicitors) or released itself by plants due to enzyme mediated action of the pathogenic microbes leading to plant cell wall degradation, intracellular proteins or small compounds formed by the plants against microbial attack or stress situations (endogenous elicitors), whereas, abiotic elicitors are non-living in nature and are divided into physical factors, chemical agents and hormones. Non-biological elicitors commonly lead to the production of plant bio actives by affecting them. But, in-vitro techniques their effect on the plant cell culture is less evident in comparison to the biotic elicitors [5, 10, 11]. Figure 2 illustrates the classification of elicitors.

###  Physical elicitors

#### Illumination

Light is a physical component by which plant bio active production is affected. Synthesis of gingerol and zingiberene synthesis was triggered on exposing *Zingiber officinale* callus culture to light. On the production of anthocyanin reaction of light irradiation was seen in *Perilla frutescens* suspension culture. A remarkable upsurge in synthesis of vinblastine and vincristine was reported when *Catharanthus roseus* plants, were exposed to UV–B light, which have resulted efficacious in the treatment of leukemia and lymphoma [4]. In *Linum album* red light was employed for podophyllotoxin synthesis [11]. Light was reported to stimulate growth of root culture and synthesis of alkaloid in *Catharanthus roseus*. Production of artemisinin in *Artemisia annua* hairy root culture was affected by light irradiation. Accumulation of phenolics like flavonoids and glucosinolates was triggered by ultra violet radiation (UV-B) which causes changes in secondary metabolism of plants. It causes an increase in nitric oxide (NO) synthesis, activities of nitric oxide synthase and phenylalanine ammonia lyase that leads to the formation of flavonoid and flavonoid level in *Ginkgo biloba* callus. For the enhancement of stilbene synthesis in distinct genotypes of grapes callus cultures irradiation with UV-C was effective [12].

#### Osmotic pressure and salinity

Osmotic stress is a non-biological physical elicitor which can change the characteristics related to the physiology and biochemistry of plants and also involved in increasing secondary metabolites concentration in plant tissues [4]. The growth, development, maturation of plant as well as secondary metabolites production is affected due to osmotic pressure. For the initiation of water stress in plants sucrose is considered to be an effective osmotic agent and is an important source of carbon and energy. In cell suspension cultures of *Capsicum chinensis* storage of capsaicin was enhanced due to influence of osmotic pressure. It also increases the steviol glycosides synthesis in callus and suspension culture of *Stevia rebaudiana* [11, 12]. Plants have developed combination of mechanisms and defensive tactics for their adaptation against stresses caused by variation in osmolarity. The formation of secondary bio active products like phenols, terpenes and alkaloids are stimulated by disclosure of plants towards salinity. In the plant cultures of *Bacopa monnieri* storage of bacoside A increased because of induction of stress by potassium chloride and calcium chloride. The production of vinblastine and vincristine in embryogenic plant tissue culture (EPTC) of *Catharanthus roseus* was reported to be ameliorated with use of chemical elicitor, NaCl [12].

####  Drought

Drought is considered as another abiotic stress which restricts growth of the plant, affects its reproductive development and on the whole its survival. Drought stress tolerance is seen in almost all plant types, however, the capacity to resist the water deficit conditions varied among different plant species. The formation as well as accumulation of plant natural products is affected by drought stress faced by the plant species. Little water stress considerably enhances the amount of the anti–inflammatory saikosaponins in *Bupleurum chinense*. Roots of *Salvia miltiorrhiza* have shown to produce increased amount of salvianolic acid on getting moderate water stress. In *Prunella vulgaris* the synthesis of various acids like rosmarinic, ursolic, and oleanolic acid was risen by slight drought stress. The glycyrrhizic acid amount in roots of *Glycyrrhiza uralensis* was reported to be uplifted by slight water deficit [4]. When the in-vitro grown date palm callus was elicited with polyethylene glycol (PEG), stimulation of drought stress occurred, the concentration of proline increased in the culture with increasing amount of PEG [11, 12].

#### Temperature extremities

Variation in temperature is considered another aspect that limits growth and decreases productivity of plant. Along with this, it alters the metabolic processes which includes, the formation as well as degeneration of plant primary metabolites. Normally, for commencing the growth of mass of cells temperature range (17–25 ± 2 °C) was suitable [12]. The incubation of culture cells of *Melastoma malabathricum* at a low temperature (20 ± 2 °C) led to enhancement in the growth as well as synthesis of anthocyanin as compared to cultures grown at higher temperatures 26 ± 2 °C and 29 ± 2 °C, respectively [12]. The amount of anthocyanin increased at an optimum temperature of 25 °C in cultured cells of strawberry and *Perilla frutescens*. In the roots of *Panax quinquefolius* the ginsenoside concentration increases with a rise of 5 °C in the temperature [12]. Changes in temperature induces several alterations in plant system such as disintegration of membrane and membrane protein complexes and protein denaturation. Furthermore, the response of plants towards temperature changes is quiet rapid that in a time span of half an hour metabolic response can happen. The pathways associated with plant responses to thermal stress are not clearly defined. However, for stimulating pathways related to heat stress transcriptional factor such as heat shock transcription factor plays a pivotal role. The low temperature conditions also cause an impact upon the synthesis of plant secondary metabolites [13]. Cold stress or low-temperature conditions such as chilling, freezing drastically affect developmental stages of plants. Temperate plant species are less sensitive to cold stress in comparison to tropical and sub-tropical plant species. Zhao et al. reported that against the low temperature condition like exposure at − 5 °C for a duration of 4 h the tea plants (*Camellia sinensis*) accumulates more of the glycosylated sesquiterpene and nerolidol glucoside, the storage was expedited by an enzyme plant glycosyltransferase, UGT91Q2) [14].

### Chemical elicitors

Chemical elicitors such as metals are known to change the secondary metabolite synthesis by bringing alterations in secondary metabolism. Metals are considered to be a major non-biological stress inducers for living entities with the advancement of their use in the developing fields like Agro technics, high bioaccumulation and toxicity. In a wide array of plants, the production of plant secondary metabolic compounds has been increased with use of metals like Ni, Ag, Fe and Co [15]. The response of plants to different metallic stress varies depending upon the concentration of metal ions, type of plant species, their growth and developmental parameters. The storage of phenolics occurred in *Gynura procumbens* on exposing the plant to Cd and Cu [15]. Treatment of *Vitis vinifera* cell suspension culture with different concentrations of cobalt (5, 25 and 50 µM), also with silver and cadmium at low quantity of 5 µM each for a duration of about 4 h were found to be efficient in enhancing the productivity of phenolic compounds and also led to increase of up to 1.6 times in the level of 3-*O*-glucosyl-resveratrol as compared to control cultures [12]. In 16-days, old hairy root cultures of *Ambrosia artemisiifolia*, thiarubrine A production increased eight times when treated for 72 h with 50 mg/L vanadyl sulfate (VOSO_4_) [12]. Further, treatment of callus culture of *Camellia sinensis* with cobalt ion as an elicitor causes enhancement of about 11.9% in the production of cinnamic acid, an essential phytochemical used as an anticancer, antioxidative substance [16].

###  Hormonal elicitors

Various hormonal elicitors are involved in incrementing the expression of genes associated with different biosynthetic pathways [14]. Jasmonic acid is hormone produced within the plant system at the time of stressful condition and acts as inducer of plant bioactive compounds. Synthesis of various types of plant derived compounds such as flavonoids, alkaloids etc. has been associated with jasmonic acid. The synthesis of soyasaponin, saponin in *Glycyrrhiza glabra* been enhanced by methyl jasmonate. By treatment with jasmonic acid, the ketone benzalacetone production was induced in *Rubus idaeus* [17]. Production of an important phenolic compound, salicylic acid synthesized from phenylalanine or isocomerate and its transportation to site of action from the apoplast was assisted by changes in the pH [18]. It has been reported in *Rubia cordifolia* the amount of anthraquinone increased with the application of salicylic acid. Likewise, in *Brugmansia candida* stimulation of tropane alkaloid scopolamine post treatment with salicylic acid. Further, the terpenoid metabolism has been impacted by salicylic acid. For instance, the storage of triterpenoids, ginsenosides and glycyrrhizin increased in ginseng and licorice, respectively [17].

### Plant based elicitors

Plant based elicitors are polysaccharide and cellulose which are components of the primary cell wall of the plants. Pectin is an important branched polysaccharide and is composed of a large number of galacturonic acid-rich polysaccharides. The primary cell of plant cells is mainly composed of three main pectic components that is homogalacturonan, rhamnogalacturonan-I and rhamnogalacturonan-II. Maximum amount of pectin is present in the middle lamella followed by decreasing concentration through the outer cell wall toward cytoplasmic membrane of plant cell [19]. Table 1 provides a few examples of plant derived elicitors.

**Table 1:** Provides a few examples of plant derived elicitors

Sr. No	Elicitors used	Production system	Secondary metabolite produced
i.	β-linked glucopyranosyl	*Glycine max*	Phytoalexins [11]
ii.	α-1,4-oligogalacturonide	*Glycine max*	Phytoalexins [11]
iii.	Chitosan	*Nicotiana tobaccum, Eschscholzia califomaica*	Phytoalexins [11]
iv.	Poly galacturonic acid	*Morinda citrifolia*	Anthraquinone [20]
v.	Oligo galacturonic acid	*Panax ginseng*	Saponin [21]

### Fungal derived elicitors

For the induction of defense responses in the plants the elicitors produced by the microorganisms are used. For the initiation of the phenylpropanoid/ flavonoid pathways in plant culture system infectious and non-infectious fungal extracts are in use [4]. The elicitors derived from fungal extracts such as Polysaccharide fraction, chitosan, fungal derived protein, mycelia homogenate and culture filtrate are pathogenic to the plants. In hairy root culture of *Ambrosia artimissiifolia* thiarubrine production was enhanced by 3 times by using fungal elicitor, *Protomyces gravidas.* The synthesis of beta lain increased by 2.6 times on eliciting hairy root culture of *Beta vulgaris* with powdery extract of fungus, *Penicillium notatum* [11]. Likewise, the polysaccharide and mycelial portion of an endophytic fungus, *Trichoderma atroviride* D16 was applied for eliciting *Salvia miltiorrhiza* hairy root culture. Polysaccharide fraction reported to have promontory effect on hairy roots development and induction of tanshinone formation by effecting the genetic expression related to metabolic pathway of plant products. A promotive effect on accumulation of azadirachtin, a complex tetra triterpenoid limonoid was recorded with the inclusion of fungal filtrate culture of *Curvularia lunata* in the *Azadirachta indica* hairy root culture, with total production of 7.1 mg/g in comparison to control cultures (3.3 mg/g) [22].

### Yeast derived elicitors

Since, a long-time yeast extract was used in elicitation by a number of researchers as a biotic elicitor. The tanshinone synthesis occurred on treating the root culture of *Perovskia abrotanoides* with yeast derived extract [4]. Application of hairy roots of *Pueraria Candollei* var. *mirifica* with yeast extract, formation of deoxymiroestrol (a phytoestrogen) and iso flavonoids takes place, while, the amount being produced depends upon the concentration of yeast extract added. According to Zhao et al. Treatment of *Fagopyrum tataricum* hairy root culture with yeast derived polysaccharide, the phenylpropanoid pathway is triggered that leads to the formation of rutin and quercetin (flavonoids) as well as have promotive influence on the growth and development of roots. With inclusion of yeast polysaccharide at an amount of 200 mg/L showed about 2.1 times increase in the overall amount of rutin and quercetin [22].

### Bacterial derived elicitors

Elicitors obtained from cell wall of bacteria and from homogenous culture filtrate of bacterial cells. These elicitors are being utilized in synthesis of plant-based substances. An increase of about 1.38-fold was reported in the beta lain formation when treatment with dry cell powder of *Lactobacillus casei* was given [11]. In the taxane media cell cultures the considerable production of taxane occurs with elicitation of cell culture with coronatine, a phytotoxin released by *Pseudomonas syringae*, a bacterial specie. In addition to this, it also stimulates viniferins formation in grape cell cultures. The root culture of *Scopolia parviflora* when treated with bacterial extract showed production scopolamine due to inhibitory effect of elicitor on gene (H6H- Hyoscyamine 6β- hydroxylase) expression [4]. Zhao et al. [22] investigated a positive effect on tanshinone accumulation (7-folds) and biomass productivity (13.6 g/L) in comparison with cultures taken as control when a culture of *Salvia miltiorrhiza* in exponential growth phase was elicited with polysaccharide-protein fraction (100 mg/L) obtained from rhizobacterium *Bacillus cereus.* Overall, enhancement in the hairy roots of amount of tanshinone was 10 times when compared with un elicited cultures of hairy root. In stationary phase of *Scutellaria lateriflora* root culture, storage of wogonin, a dihydroxy- and monomethoxy‐flavone was enhanced by around 30 mg/g when culture media was employed with *Pectobacterium carotovorum* lysate [22].

## Criterion of elicitors

Elicitation has been broadly used in enhancing the synthesis or triggering the production of phytochemicals in-vitro culture conditions. Numerous factors like amount of elicitor, specificity of elicitor, duration of exposure, regulation of growth, nutritional composition, and quality of substances being used are some of the parameters which plays an essential role in determination of plant secondary metabolite productivity. A certain number of the factors are discussed below.

### Elicitor concentration

In elicitation process the concentration of elicitor is playing a very crucial role. Hypersensitive response is stimulated in the plants which leads to cell death when a dosage more than optimum is given, however for induction of secondary metabolite synthesis optimum concentration of elicitors is required [4, 10]. On application of salicylic acid, the productivity of ginseng and saponin were enhanced to about 1.15 and 1.13 times, respectively as compared to cultures taken as control with inclusion of 0.1% of NaCl to the culture media [23]. Different concentrations such as 50, 100, 150, 200, and 250 µM of methyl jasmonate and salicylic acid were added to the suspension culture of *Gymnema sylvestre*. It was investigated that storage of gymnemic acid increased when 150 µM and 200 µM amount of methyl jasmonate and salicylic acid were used, individually [24]. The phenolic acid formation was triggered in the *Vitis vinifera* cell suspension culture when cobalt at different amounts (5.0,25,50 µM), silver and cadmium at low concentration of 5.0 µM separately, were used [25]. Table 2 summaries the elicitors, the concentration of elicitors for producing the commercially valuable plant product, amount of the metabolite produced through in-vitro culture system in different plant species.

**Table 2:** Summaries the elicitors, the concentration of elicitors for producing the commercially valuable plant product, amount of the metabolite produced through in-vitro culture system in different plant species

Sr. No	Elicitor	Plant species	Culture system	Elicitor conc.	Type of PSM	Plant product	Increment in product yield in comparison to control	References
1.	Yeast extract	*Scutellaria lateriflora*	HRC	50 µg/mL	Phenylethanoid glycoside	Acetoside	1.4-fold	[26]
2.	Fungal mycelium of *Botrytis* sp.	*Papaver somniferum*	Cell culture	1 mL	Alkaloid	Sanguinarine	26-fold	[26]
3.	NaCl	*Solanum khasianum*	HRC	100 mM	Alkaloid	Ajmalicine	14.8-fold; 0.058 mg/g DW	[27]
4.	Chitosan	*Lepidium sativum*	Callus culture	250 mg/L	Imidazole alkaloid	Lepidin	19.87-fold	[28]
5.	GA3	*Lepidium sativum*	Callus culture	2 mg/L	Imidazole alkaloid	Lepidin	3.89-fold	[29]
6.	Cell extract of *Coniothyrium palmarum*	*Coryeus avellane*	CSC	5% (v/v)	Diterpenoid	Paclitaxel	3.9-fold	[30]
7.	Methyl jasmonate	*Valeriana officnalis*	HRC	100 µM	Sesquiterpenoid derivative	Valerenic acid	6-fold	[31]
8.	Methyl jasmonate	*Panax ginseng*	Adventitious root culture	100 µM	Ginsenosides	Ginseng	8-fold; 26.6 mg/g DW	[32]
9.	Chitosan	*Iberis amara*	Callus culture	50 mg/L	Phenolic compounds	Phenol	34.1 mg/g DW	[33]
10.	Chitosan	*Barringtonia racimosa*	CSC	150 mg/L	Phenolic compounds	Gallic acid	1.3-fold	[34]
11.	Jasmoninc acid	*Bacopa monnieri*	In vitro shoot culture	1 mg/ L	Saponins	Bacosides	3.08-fold;8.46 mg/g DW	[35]
12.	Methyl jasmonate	*Bacopa monnieri*	In vitro shoot culture	50 µM	Saponins	Bacosides	1.8-fold; 4.4 mg/g DW	[35]
13.	CuSO_4_	*Bacopa monnieri*	In vitro shoot culture	45 mg/L	Saponins	Bacosides	1.42-fold; 8.73 mg/g	[35]
14.	Methyl jasmonate	*Artemisia annua*	SC	5 mg/L	Sesquiterpene lactone	Artemisinin	76.03 mg/L	[36]
15.	Salicylic acid	*Stephania venosa*	SC	2 mg/L	Iso quinoline	Decentrine	22.4 mg/g DW	[37]
16.	Methyl jasmonate	*Taxus media*	CSC	100 µM	Diterpenoid	Paclitaxel	5.1-fold	[38]
17.	Salicylic acid	*Cayratia trifolia*	CSC	500 µM	Stilbenes	Resveratrol	68 µg/g	[39]
18.	Salicylic acid	*Cayratia trifolia*	CSC	500 µM	Stilbenes	Viniferin	223 µg/g	[39]
19.	Salicylic acid	*Cayratia trifolia*	CSC	500 µM	Stilbenes	Ampelopsin	958 µg/g	[39]
20.	*Bacillus cereus* culture	*Salvia miltiorrhiza*	HRC	0.2% v/v	Diterpenoid	Tanshinone	12-fold	[40]
21.	AgNO_3_	*Ocimum basilicum*	CSC	25 µM	Terpenoid	Linalool	4.37 µg/g DW	[41]
22.	AgNO_3_	*Ocimum basilicum*	CSC	5 µM	Terpenoid	Estragole	3.30 µg/g DW	[41]
23.	Jasmonic acid	*Calendula officinalis*	HRC	100 µM	Saponins	Oleanolic acid	28.46 mg/L	[42]
24.	Salicylic acid	*Isatis tinctoria*	HRC	142.61 µM	Alkaloid	Indigo	3.15 mg/g	[43]
25.	*Piriformospora indica* culture filtrate	*Lantana camara*	CSC	2.5% v/v	Pentacyclic triterpenoid	Betulinic acid	7.8-fold; 117.02 µg/g DW	[44]

###  Exposure time to elicitor

When the treatment of methyl jasmonate was given to cell suspension culture of *Gymnema sylvestre* for different durations (24 h, 48 h, 72 h) with a concentration of 150 µM the highest biosynthesis of gymnemic acid (135.41 ± 0.43 mg/g dry cell weight) was reported at time period of 72 h. The concentration of gymnemic acid in elicited cultures were 15.4 times more in comparison to the cultures without elicitors [24, 45]. To stimulate the content of gymnemic acid in cell suspension culture of *Gymnema sylvestre* different types of fungal and bacterial preparations (*Agrobacterium rhizogenes*, *Bacillus subtilis*, *Escherichia coli*, *Aspergillus niger*, and *Saccharomyces cerevisiae*) at distinct time periods (24, 48, 48, 72, and 72 h, respectively) were used with highest amount reported with *Aspergillus niger* after 72 h [46]. The shoots culture of *Bacopa monnieri* elicited with methyl jasmonate synthesized maximum content of bacoside compounds, a concentration 1.5 times more than the control cultures after 2 days [47]. In the hairy root culture of *Datura metel* amount of atropine enhanced to different levels after 12 h, 24 and 48 h when Nano silver was added in to culture media [48]. The cell culture of *Catharanthus roseus* was treated with 5% filtrates of fungus like *Trichoderma viride, Aspergillus niger and Fusarium moniliforme*, respectively for different time durations of 24 h, 48 h, 72 and 96 h. The synthesis of ajmalicine enhanced by 3 times in cell culture elicited with *Trichoderma viride* for duration of 48 h, while, culture to which *Aspergillus niger* and *Fusarium moniliforme* biotic elicitors were added 2-fold rise was recorded [49]. The hormonal elicitor, methyl jasmonate (5 µM) stimulated maximum storage of andrographolide compounds at 24 h as compared to 48 and 72 h in *Andrographis paniculate* cell culture [50].

### Culture age

Another major factor involved in formation of plant secondary metabolites is culture age. The maximum storage of withanolide A, withanone, and withaferin A compounds occurred in *Withania somnifera* hairy root culture on eliciting with salicylic acid after 40 days of culturing [51]. Productivity and amount of ginseng and saponin enhanced to approximately 1.31 and 1.33 times in comparison to the control when to 21 days old culture a heavy metal elicitor, selenium (0.5 mM) was added [23]. With the introduction of methyl jasmonate to suspension cultures of *Catharanthus roseus* (6 days old) at a concentration level of 10 µM and 100 µM rose the synthesis of ajmalicine and serpentine, discretely. However, antagonistic effect on the growth and alkaloid formation was observed on re-elicitation [52]. An elicited 20 days old culture of *Catharanthus roseus* produced higher amounts of bio active ajmalicine. The 20 days old culture reported to have the largest accumulation of ajmalicine (166 µg/g of dry weight) when treated with the fungal extracts of *Trichoderma viride*, whereas, the cell culture elicited with the exogenous extracts of *Aspergillus niger* and *Fusarium moniliforme* recorded to have accumulated 90 µg/g and 88 µg/g of dry weight of ajmalicine compounds, respectively [10, 49].

### Nutritional makeup

Composition of medium or media selection are considered another important factor in process of elicitation. The quantities of different secondary metabolites such as cocaine, cinnamoyl cocaine, chlorogenic acid (CGA), and 4-coumaroyl quinate (CQA) were remarkably influenced by constituents of culture medium of *Erythroxylum coca* callus culture [53]. The synthesis of cocaine was reported to be almost similar on culture medium such as Gamborg B5, Murashige-Tucker medium. With regard to chlorogenic acid formation, the media were quite dissimilar from each other with maximum synthesis on ARM and minimum on MMT. On ARM media 4-coumaroyl quinate production was less in comparison to other two media (Gamborg B5, Murashige-Tucker medium) which were almost similar in their nutrient composition. The main parameter responsible for checking the accumulation of tropane (an alkaloid) was composition of nutrient media. The content of tropane production was investigated to be higher on using ARM as the culture media due to the presence of different components in ARM media as compared to other media [53]. Further, in the literature there were many reports that showed a connection between nitrate availability and secondary metabolite storage to be inversely related to each other in a number of different plants belonging to distinct species like *Arabidopsis thaliana* [54], *Hordeum vulgare* [55], and *Nicotiana tabacum* [56]. Likewise, decreased concentration of nitrate in hairy root culture of *Atropa belladonna* showed enhancement in the amount of an alkaloid as compared to the culture grown in the general Murashige and Skoog media [57]. Besides these characteristics, elicitation efficiency is affected by the specificity of elicitor, presence of PGRs, medium composition, the cell lines or clones of microbial elicitors and environmental conditions provided [4, 10].

## Common mode of action of elicitors

At less concentration, elicitors act as signaling molecules and therefore, provides information to plants for stimulating the defense responses [58]. In every plant species there exist a defense response system that have the capacity to respond against attack of pathogens, elicitors or any kind of unfavorable environmental conditions. The genetic makeup as well as the physiological state of the plants determines the defense response delivered by the plant species. Generally, the resistance of the plants against different diseases is due to the presence of plant resistance genes and pathogen avirulent a virulence gene [11]. The complementarity that exist between gene pairs of particular pathogenic race and host determines gene-for-gene interaction [58].

**Fig. 3:**
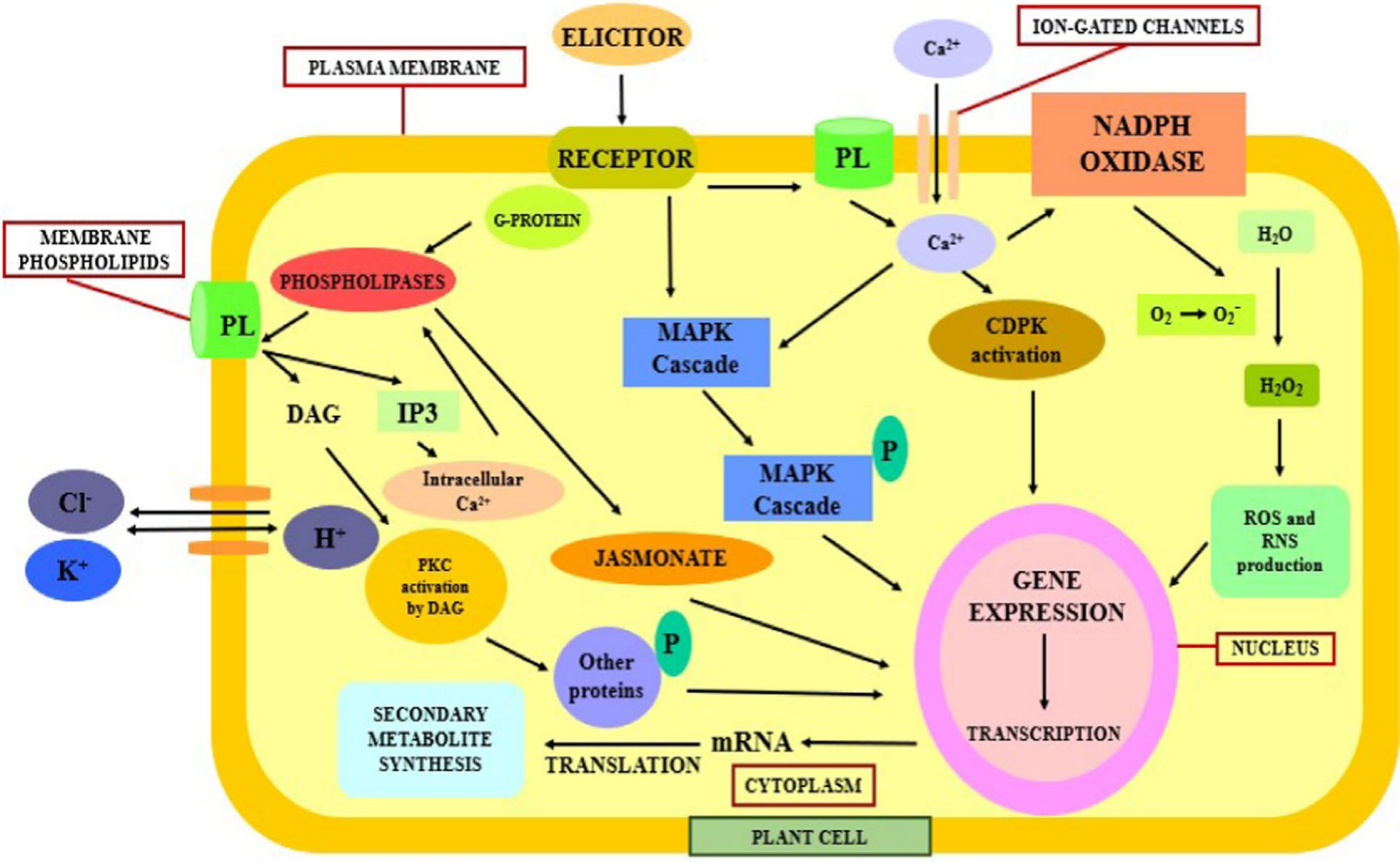
Illustrating ubiquitous mechanism of action of elicitors inside of a plant cell. CDPK Calcium dependent protein kinases, MAPK Mitogen activated protein kinase, DAG Diacylglycerol, IP3 Inositol triphosphate, PKC Protein kinase C

Although, there is a wide range of receptors present inside the cytoplasm of the cell, on the surface of the cell membrane as well as inside the nucleus. However, the receptors associated with the cytoplasmic membrane are briefly described here since these types of receptors have been broadly investigated in the plant culture system [5]. In the different plant species, different receptors interact with the elicitors which can further induce signals within the cell. The proteins of the plasma membrane and cytosol undergoes reversible phosphorylation and dephosphorylation. Efflux of chloride ion and potassium ion from the cell and in flow of hydrogen ions leading to extracellular alkalization and intracellular acidification. Activation of mitogen-activated protein kinase (MAPKs) cascade by G-protein coupled with membrane receptors that are sequentially activated further via phosphorylation. Further, influx of the calcium ions into the cytosol of the plant cell happens. Activation of NADPH oxidase and formation of reactive oxygen species as well as reactive nitrogen species induced by calcium ions. Likewise, calcium ions are also responsible for stimulating calcium dependent protein kinases (CDPK) that plays a role in regulating the biosynthesis pathways. Production of plant bio active compounds by the expression of functionally active genes. Signaling molecules such as salicylic acid, jasmonate etc. that are produced by phospholipases activate de-novo bio synthesis of transcriptional factors which controls the gene expression involved in enzyme production and ultimately, the secondary metabolites formation [5, 45]. Figure 3 depicts the ubiquitous mechanism of action of elicitors inside of a plant cell. Table 3 provides information about enhancement of the biosynthetic pathways involved in production of plant product in-vitro culture system of different plant species using elicitors.

**Table 3:** Summaries the elicitors, enhancement of the biosynthetic pathway involved in production of plant product in-vitro culture system of different plant species

Sr. No	Elicitor	Plant species	Culture system	Pathway enhanced	Type of PSM	Plant product	References
1.	Yeast extract	*Salvia castanea* Diels f. *tomentosa*	HRC	Tanshinone biosynthetic pathway	Diterpene quinone	Crypto Tanshinone	[22]
2.	Yeast polysaccharide	*Fagopyrum tataricum*	HRC	Phenyl propanoid pathway	Flavonoids	Rutin and Quercetin	[22]
3.	UV-B	*Fagopyrum tataricum*	HRC	Flavonoid biosynthetic pathway	Flavonoids	Rutin and Quercetin	[22]
4.	Silver ions	*Salvia castanea* Diels f. *tomentosa*	HRC	Tanshinone biosynthetic pathway	Diterpenoid	Tanshinone	[22]
5.	CHI	*Isatis tinctoria*	HRC	Flavonoid biosynthetic pathway	Flavonoids	Quercetin	[22]
6.	Yeast extract	*Agastache rugosa*	CSC	Phenyl propanoid pathway	Phenyl propanoid	Rosmarinic acid	[59]
7.	Silver nitrate	*Agastache rugosa*	CSC	Phenyl propanoid pathway	Phenyl propanoid	Rosmarinic acid	[59]
8.	Methyl jasmonate	*Centella asiatica*	CSC	Centelloside biosynthetic pathway	Triterpene saponins	Centelloside	[60]

## Future prospects

With the use of molecular biotechnology-based strategies, the productiveness of plant in-vitro system could be augmented. The novel field of synthetic biology has gained pace with enhancement in technology resulting in better understanding about the pathways related to the synthesis of secondary metabolite in combination with the changes in the microbial system [61]. The approach of manipulating the genes have led to the improvement in the production of pharmaceutically important molecules in-vitro conditions [62]. Metabolic engineering is considered to be an important enzymatic process dealing with the formation of new molecules within the system of a living organism [63]. Metabolic engineering has paved a new way towards enhancing productivity of plant based natural products by altering pathways associated with the synthesis of pharmaceutical important biomolecules. Further, the overexpression of genes responsible for formation of pathway regulatory enzymes upsurge the phytochemical production [64]. In addition to this, reconstruction of the biosynthetic pathways using approaches such as metabolic engineering and synthetic biology for heterologous expression of plant genes into microbial system could increase the production of phytochemicals and could also led to the formation of novel bioactive compounds. To exemplify, the production of amorphadiene and dihydroartemisinic acid has risen up by engineering the mevalonate pathway in yeast, *Saccharomyces cerevisiae* by the overexpression of enzymes associated with the pathway [65]. The yield of phenylpropanoids, naringenin and resveratrol obtained was 7 mg/L and 300 µg/L respectively, in *Saccharomyces cerevisiae* [66]. The technology of ‘omics’ whether genomics, transcriptomics, proteomics or metabolomics has been used in knowing the genetic make-over of an organism. Transcriptome profiling of an organism and identification of genes responsive towards production of plant secondary metabolite can be obtained through microarray assay [63]. For instance, rice cell suspension culture (RCSC) treated with abscisic acid (ABA) inspected for real time proteomics and metabolomics profiling helped in evaluating the effect of the physicochemical conditions [67]. Likewise, using transcriptomic analysis *Taxus* cell culture was evaluated for genes associated with enzymatic synthesis related with taxane production [68]. The rosemary cell suspension culture was evaluated for differentially expressed genes associated with synthesis of bioactive compounds through transcriptomic studies [69]. In addition to this, identification of genes along with intermediary compounds associated with the formation of camptothecin from *Ophiorrhiza pumila*, cell suspension and hairy root culture was investigated using transcriptomic and metabolomic analysis [45].

The manipulations into the plant natural product (PNP) forming biosynthetic pathways require a competent host organism, desired route expression into the system and identifying enzymes involved in the biosynthesis [70]. In addition to this, for metabolic engineering of plants, factors associated with regulation and effect upon changes in physiological as well as metabolic channeling needs to be considered. For high throughput plant derived products production using via in-vitro tissue culture system, transference of targeted gene by *Agrobacterium* transformation and alterations into the enzymatic pathways has been a commonly deployed approach. For instance, rosmarinic acid formation in various hairy root cultures have shown an increment by gene (SmC4H & SmTAT) cloning from *Salvia miltiorrhiza*. A few more examples to cite, the introduction of a coding genes for an enzyme hyoscyamine 6β-hydroxylase into *Atropa belladonna* from *Hyoscyamus niger* caused conversion of hyoscyamine into scopolamine. Furthermore, addition of the same gene has led to 100 times enhanced tropane, an alkaloids formation in *Egyptian henbane*. Overexpression of the gene ORCA3, from *Catharanthus roseus* led to increase in the amounts of alkaloids in the desired cell lines [62]. In the leaf cells of *Nicotiana tabacum*, artemisinic alcohol at a concentration of 0.01 mg/g dry weight was produced due to the expression of amorpha-4,11-diene synthase, amorphadiene monooxygenase, aldehyde D reductase and aldehyde dehydrogenase [71]. In the hairy root culture of *Campanula medium* polyacetylene, an alkaloid has been formed by *Agrobacterium* transformation method. Using the same metabolic engineering strategy various essential oils such as polyphenols, glycosides, anthraquinones etc. have been synthesized. Addition of two genes into hairy root culture of *Anisodus acutangulus* has caused 8.7 times increase in alkaloid accumulation. Furthermore, in suspension cultures of *Catharanthus roseus*, the upregulation of transcriptional factors ORAC2 or ORAC3 leveled up the formation of phytochemicals such as serpentine, tryptamine, ajmalicine etc. Alternations in the phenylpropanoid pathways has shown to upsurge the codeine and morphine production in opium poppy [66]. In *Nicotiana tabacum* cell suspension culture simultaneous expression of transcriptional factors MYB Rosea1 (AmRos1) and bHLH Delila (AmDel) from *Antirrhinum majus* led to stable synthesis of anthocyanin [71]. The quantity of tryptophan augmented by 25 times by transforming *Bacopa monnieri* with tryptophan-decarboxylase and strictosidine-synthase genes obtained from *Catharanthus roseus* using *Agrobacterium tumefaciens*. Using *Agrobacterium tumefaciens* for incorporating tryptophan-decarboxylase and strictosidine-synthase genes into *Catharanthus roseus* have caused increment in the synthesis of an alkaloid, terpenoid indole due to the upregulation of the genes [72].

The metabolic engineering has been revolutionized with emergence of genome editing techniques like zinc finger nucleases (ZFNs), transcription activator-like effector nucleases (TALENs), tetratricopeptide repeat proteins and clustered regularly interspaced short palindromic repeat (CRISPR) [73, 74]. The genome editing technology in combination with plant tissue culture and genetic transformation has led to improvement of different crop plants [75]. Among various editing techniques available, CRISPR, a simple, easy to apply tool based upon DNA–RNA interaction [66] is a dominantly applied editing approach enabling the alterations into biosynthetic pathways, quality enhancement and upgradation of the considerable essential plant natural products of pharmaceutical and therapeutic importance [65]. As for this powerful tool requirement of any specific protein for combining with the DNA is not there, as in case of other techniques such as TALENs and ZFN [75]. For the plant species, the commonly used CRISPR systems include CRISPR-cas9 and CRISPR-Cpf1. The technique is highly efficacious in context of introduction of multiple gene mutations within the plant systems and understanding the functioning of specific metabolic enzymes. The interaction among the genes involved in biosynthetic pathways obtained via CRISPR tool could be analyzed through metabolic profiling [73]. To exemplify, CRISPR-cas9 has been employed in knocking out a gene (SmCPS 1) in *Salvia miltiorrhiza* that declined the production of tanshinone. Likewise, in opium poppy the synthesis of benzylisoquinoline alkaloids (BIAs) dropped considerably due to knocking out of 4 OMT2 gene [66]. The technique of CRISPR/Cas has been used for stopping the production of anthocyanin in the carrot cells by targeting the flavanone-3-hydroxylase gene. For improving the quality of recombinant IgG in *Nicotiana tabacum* BY-2 cells CRISPR multiplexing was done on two β-(1,2)-xylosyl transferase (*XylT*) genes and four α-(1,3)-fucosyl transferase (*FucT*) genes respectively. Furthermore, addition of heterologous genes in rice cell suspension culture (RCSC) applied through CRISPR/Cas mediated insertion/replacement led to the production of novel Phyto chemicals [67]. In addition to this, knocking out of the rosmarinic acid synthase gene and diterpene synthase gene in *Salvia miltiorrhiza* has shown a decline in the formation of rosmarinic acid and lithospermic acid B and tanshione [76]. The amount of morphine and thebaine declined and benzylisoquinoline, an alkaloid a novel bioactive compound has been produced in Papaver somniferum by knocking out 3′-hydroxyl-*N*-methylcoclaurine 4′-*O*-methyltransferase (4′ OMT2) using CRISPR/Cas9 [62].

However, these technologies suffer from challenges that can be worked upon allowing the successful production of commercially valuable plant products [77]. For instance, complex metabolic pathways make it troublesome for reconstruction of plant biosynthetic pathways in heterologous microbial systems such as multiple P450 reactions are involved in the production of paclitaxel. Further, low functioning efficiency of plant derived enzymes into alterative host system and toxicity in product formed could also be there. In addition to this, longer growth cycle of plants possesses another hindrance in plant secondary metabolite synthesis [71]. Although, CRISPR-Cas, a genome editing tool has led to many developments in the field of plant sciences yet it has limitations such as stable transformations and regeneration of edited plants which could be researched upon for the optimization of the protocols [73]. Therefore, overcoming the bottlenecks associated with approaches of metabolic engineering could the approach a promising one for future developments in pharmaceutical based industries.

## Conclusion

Plants have long been considered a substantial source for several plant metabolic compounds. This is due to the remarkable biological qualities and traits that these plant bioactive chemicals have, which are extremely significant for the pharmaceutical sector. Plant tissue culture techniques have been quite useful to improve production of medically valuable products via elicitation process. One of the major hurdles in phytochemical synthesis using elicitation approach is finite knowledge regarding the highly complex bio synthetic pathways and genes and enzymes regulating them. Along with this, absence of elucidation of the compatible transcription factors and master regulators are additional factors. Therefore, an in-depth understanding about the response to biological and non-biological plant stressors in tissue culture and their specific secondary bio active pathways is required. The study of proteomics and metabolomics can un reveal the elicitation of plant bio actives and their relationship with primary metabolites. Furthermore, with deeper understanding of the metabolic pathways, the exact application of elicitor-driven effects onto the selected plant cell culture may become a successful approach in obtaining cell cultures with high productivity. Nonetheless, for accomplishing higher yield, even cell cultures, which are engineered for overexpressing some key genes of selective biosynthetic pathways, need elicitation. Hence, for plant cell culture selecting a suitable elicitor will remain critical. However, for the ultimate development in a cost effective, large scale production of plant secondary metabolites will depend on better understanding about the metabolic responses to elicitation in plant cell culture and the mechanisms accountable for these effects. Recent advancement in synthetic biology such as modification in biosynthetic pathways, overexpression of genes involved in synthesis of bioactive components, editing the plant genome via CRISPR-Cas genome editing tools etc. has enabled the formation of highly valuable added secondary compounds to become a reality. However, problem of production of stable transformants, functional inefficiency of plant system-based enzymes in alternate host, complexity of pathways associated with synthesis of plant secondary metabolites etc. linked with these methodologies need to be researched upon for upgrading the production of plant based natural products for fulfillment of the demands on the commercial scale.

## Data Availability

Not applicable.
